# Increasing smoking cessation care across a network of hospitals: an implementation study

**DOI:** 10.1186/s13012-016-0390-x

**Published:** 2016-03-01

**Authors:** Carolyn Slattery, Megan Freund, Karen Gillham, Jenny Knight, Luke Wolfenden, Alessandra Bisquera, John Wiggers

**Affiliations:** 1 Population Health, Hunter New England Local Health District, Booth Building, Wallsend Health Services, Longworth Avenue, Wallsend, NSW 2287 Australia; 2 Hunter Medical Research Institute, Clinical Research Centre, Lot 1 Kookaburra Circuit, New Lambton Heights, NSW 2305 Australia; 3 School of Medicine and Public Health, Faculty of Health and Medicine, The University of Newcastle, University Drive, Callaghan, NSW 2308 Australia

**Keywords:** Smoking cessation, Hospital, Nicotine replacement therapy, Practice guidelines

## Abstract

**Background:**

Despite clinical practice guidelines recommending the provision of smoking cessation care to all smokers in hospital, the provision of such care can be sub-optimal. A study was conducted to assess the impact of an intervention on the provision of smoking cessation care to nicotine-dependent smokers across a network of hospitals.

**Methods:**

A 4-year interrupted time series study was undertaken in a single health district in New South Wales, Australia. A multi-component intervention was implemented over a 2-year period in all 37 public general hospitals. Outcome data were collected from eight randomly selected hospitals via medical record audit. Logistic regression analyses assessed differences between baseline, intervention and follow-up periods in the provision of seven measures of care: brief advice, offer and provision of inpatient and discharge nicotine replacement therapy, and offer and acceptance of referral to a Quitline.

**Results:**

Approximately 164,250 patients were discharged from the hospitals during the study, 16 % of whom were smokers. Of the selected smokers, 56.12 % (*n* = 2072) were nicotine-dependent. The prevalence of smoking cessation care increased significantly for all seven measures between baseline and intervention periods, and for six of the seven measures between the baseline and follow-up periods. The odds of receiving care at follow-up were between 1.7 (CI 1.18–2.58, *p* = 0.0004) and 6.2 (CI 2.84–13.85, *p* < 0.0001) times greater than at baseline. At follow-up, 53, 16 and 7 of smokers were offered inpatient NRT, discharge NRT and a Quitline referral, respectively.

**Conclusions:**

Significant gains in the provision of smoking cessation care were indicated. However, at best, slightly more than half of the patients received smoking cessation care. Additional care enhancement strategies are required if all smokers are to obtain the intended benefits of smoking cessation care guidelines.

## Background

Despite the success of tobacco control initiatives in many countries, the prevalence of smoking remains unacceptably high [1, 2]. To reduce this burden at both the individual and population levels, smoking cessation clinical practice guidelines recommend the provision of smoking cessation care to all smokers attending health services including hospitals [3–7]. Smoking cessation programmes that begin during a hospital stay and include counselling with follow-up support for at least 1 month after discharge are effective in increasing smoking cessation. Such programmes are effective regardless of the reason for admission. The addition of nicotine replacement therapy to a counselling program increases cessation rates [8]. Cessation guidelines place a particular emphasis on the routine provision of cessation advice and counselling, pharmacotherapies (e.g. NRT, varenicline and bupropion) [8, 9] and offer of post discharge care [3, 5–7, 9].

Despite such guidelines, the provision of such care is less than optimal within the USA, Australia and other countries [10–16]. Intervention research has demonstrated that the provision of smoking cessation care in hospitals can be increased [17–19]. However, the demonstrated increases in care are often small or moderate, and hence, a significant proportion of smokers do not receive appropriate cessation care [18]. In contrast, in the USA, hospital accreditation by the Joint Commission (an independent, not-for-profit organization, that accredits and certifies more than 20,500 health care organizations and programs) has been reported to be associated with large increases in the proportion of patients provided with smoking cessation advice ranging from 37 to 67 % in 2002 to 95 to 97 % in 2008 [16]. Despite the suggestion of almost universal and sustained access to such care, interpretation of such findings is constrained by the data being reported only for those patients admitted for three specific conditions (myocardial infarction, congestive heart failure or pneumonia), addressing only one element of evidence-based smoking cessation care (provision of advice) [15, 16] and the risk of bias associated with hospital self-report of care delivery [20]. In addition, considerable variability in the provision of such care has been reported by both type of hospital and type of patient [16].

Since 2012, a broader range of smoking cessation care elements, including cessation treatment during admission and at discharge has been included in Joint Commission reporting requirements [15]. However, reporting of the delivery of such care is at the discretion of hospitals, resulting in a potential bias in estimates of care provision across hospitals [15, 16, 21]. In addition, no information is provided regarding the implementation strategies applied by hospitals to achieve increases in care delivery. This is a key gap in understanding how universal access to smoking cessation care can be achieved by all hospitals for all patients both in the USA and in other countries, particularly given the lack of accreditation standards for hospital smoking cessation care in Australia and other jurisdictions [22, 23].

Given the limitations of available evidence regarding the effectiveness of strategies in increasing the provision of recommended smoking cessation care to all hospital patients, a study was undertaken to assess the impact of a multi-component practice-change intervention on the provision of such care to smokers across a network of Australian hospitals.

## Methods

### Design and setting

A 4-year interrupted time series implementation study was conducted from 2005 to 2009 in a public Local Health District in New South Wales, Australia. Outcome data were collected for the 4-year study period (12 months baseline, 24 months intervention and 12 months follow-up).

The District provided public hospital, ambulatory and population health services to approximately 850,000 people residing in metropolitan, rural and remote communities.

Approval for the study was provided by the Hunter New England Human Research Ethics Committee.

### Participants

#### Hospitals

All inpatient wards in all 37 general hospitals that provided medical and surgical care to adult patients received the intervention. The hospitals varied in terms of their size and function and were categorised as either: Group A (tertiary referral), Group B (rural referral/acute), Group C (district), Group D (community) and Group E (multi-purpose).

For the purposes of evaluating the impact of the intervention, Group D and Group E hospitals were excluded from the study given the limited numbers of patients being discharged. Further, due to cost feasibility considerations, outcome data were collected from a randomly selected representative sample of 8 of the remaining 17 hospitals (47 %), stratified by hospital group: Group A (1 out of 1 hospital), Group B (2 out of 4 hospitals) and Group C (5 out of 12 hospitals). The random digit function in Microsoft Excel was used to randomly select the hospitals. The eight selected hospitals accounted for 63 % of all general hospital beds in hospital groups A, B and C and 53 % of all such beds in the Health District.

#### Patients

All inpatients, other than mental health, intensive care, substance detoxification or maternity patients, who were discharged from the eight selected hospitals over a 48-month period and were recorded in the electronic medical record as being a smoker [24], an inpatient for at least 24 h, and 18 years of age or over, were identified. Mental health, intensive care, substance detoxification and maternity patients were excluded from data collection, based on their diagnostic codes, as they have special needs likely to impact on the level of smoking care provided. Of these smokers, for every 2 months over the study period, approximately 14 % were randomly selected from each hospital group. Smokers could not be selected more than once in the same period. Random selection was conducted using SAS version 9 statistical software [25], using the RANUNI function.

An audit of the paper medical records of the randomly selected smokers was conducted to determine whether the patient was recorded as being nicotine-dependent. As recording of nicotine-dependence by clinicians is often not standardised, patients were classified as nicotine-dependent if in the medical record it was recorded that they smoked more than 10 cigarettes a day, or more than 160 packs per year, or more than 3 packs per week, or within 30 min of waking up in the morning or were recorded in the medical record as a ‘heavy’ smoker [26]. Such nicotine-dependent patients constituted the study sample.

### Smoking cessation care

Prior to the intervention, based on a pre-existing state-wide guideline [5], all hospital clinicians were recommended to provide the following forms of smoking cessation care to nicotine-dependent patients: nicotine-dependence assessment of all smokers; and if dependent, provision of NRT during inpatient stay; provision of brief smoking cessation advice; provision of a 3-day supply of NRT on discharge; and referral to the free NSW Quitline. The primary goal of the guideline was the effective treatment of nicotine-dependent patients in NSW Health facilities.

### Practice change intervention

A service quality improvement intervention was delivered concurrently over a 24-month period to all 37 hospitals in the context of the implementation of a state-wide smoke free policy. All clinicians and managers in the hospitals (approximately 4100) were the focus of the intervention.

Based on practice guidelines [3, 4], reviews of strategies to enhance the provision of smoking cessation care and practice change more generally [18, 27–29], a multi-component implementation intervention was delivered involving the following strategies.

#### Executive leadership and establishing consensus

The intervention was implemented as a whole-of-organisation strategic initiative. The Health District Chief Executive formally endorsed the initiative and disseminated this endorsement through senior management meetings and routine Health District newsletters, established an Implementation Committee led by two Executive Sponsors, established an implementation project team and an Executive level annual implementation review meeting. Clinical managers and clinicians developed a local smoking cessation care guideline and associated training program and care delivery tools [4].

#### Care delivery tools

The following tools to facilitate clinician provision of nicotine-dependence treatment were developed: nicotine withdrawal assessment tool; protocol for nurse-initiated NRT provision; nicotine-dependence care form to prompt offer and provision of treatment (included the Heavy Smokers Index); a bedside audit tool and a mandatory reporting tool. All tools were emailed to all nurse unit managers, made available via the Health District intranet site and addressed in training [3, 27, 28].

#### Clinician training

Four, half day train-the-trainer workshops were conducted for nursing staff representing all inpatient wards in the 37 hospitals. One hundred and twenty four nursing staff participated in the workshops delivered by a registered nurse who had conducted a New South Wales Telehealth smoking course. The training included: information on the smoke free policy, support available for staff, how the policy is communicated, monitoring, compliance and enforcement, identifying and recording a smoking incident, grievance resolution procedures, identifying and assessing nicotine-dependence, managing nicotine-dependence, prescribing NRT, monitoring withdrawal symptoms, and discharge and referral procedures relating to ongoing smoking cessation care. The trained staff subsequently delivered the training to nursing staff in their respective hospital wards [3, 4, 27].

#### Practice change support

Four tailored telephone calls were made with each Senior Nurse Manager and/or Nurse Unit Manager of each inpatient ward in all hospitals over the 24-month intervention period. The calls addressed: receipt and use of nicotine-dependence treatment tools and resources, provision of brief advice, and amendment of ward forms to include mandatory reporting requirements, promotion of the intervention support service, and identification of barriers, provision of advice, problem solving and feedback in response to monitoring reports.

#### Care delivery monitoring and feedback

Structured bedside audits were conducted for every patient in every inpatient ward in each facility on a single day on three occasions during the 24-month intervention period (3, 11 and 20 months after commencement of intervention) to monitor the delivery of nicotine-dependence treatment. The audit was conducted by a ward staff member and involved an initial review of each patient’s medical record and subsequent patient interview to ascertain whether the patient had been provided nicotine-dependence treatment during their current admission. Tailored progress reports describing the results of the bedside audits for each ward were emailed to all Nurse Unit Managers, Senior Nurse Manager/s, Cluster Managers of each ward, hospital managers and the District Implementation Committee [3, 21, 27, 29].

#### Communication

The rationale and progress of the intervention were communicated to hospital staff via electronic newsletters, broadcast emails, fact sheets and executive, management and staff meetings. The Chief Executive actively promoted smoking cessation care through newsletters and executive meetings [3, 4, 27].

### Data collection procedures

The paper medical records of the selected nicotine-dependent patients were retrospectively audited by trained clinical auditors employed by the research team. All sections of each medical record were reviewed [17]. Patient demographic and clinical information were obtained from the medical records and from patient electronic data records.

A 4 % random sample of audited patient paper medical records was reaudited to assess inter-rater reliability.

### Measures

#### Patient demographic and clinical characteristics

The following patient characteristics were collected from the electronic medical record: age, gender, Aboriginal and/or Torres Strait Islander status, hospital group, and ward of discharge, length of stay and diagnoses (smoking-related/not smoking-related) [24].

#### Smoking cessation care

Seven outcome measures addressed three types of smoking cessation care: quit advice, nicotine-replacement therapy (NRT) and referral to the Quitline.

1. Quit advice
  Quit advice was classified to have been provided if there was a notation of a patient being provided with advice to quit (e.g. ‘patient advised to stop smoking’) (yes, no).
2. NRT
*Offer of inpatient/discharge NRT*. Patients were classified to have been offered NRT if there was any notation of NRT provision in the medication list, or in the medical record/discharge summary of the patient (e.g. ‘patient offered NRT’); or any notation of the patient accepting, refusing or being provided NRT (yes, no).
*Provision of inpatient/discharge NRT.* Patients were classified to have been provided NRT if there was any notation of NRT provision in the medication list, or in the medical record/discharge summary (e.g. ‘patient provided with 3-day supply of NRT to take home’) (yes, no).
3. Quitline Referral
*Offer of referral to Quitline.* Patients were classified to have been offered referral to Quitline if there was any notation in the medical record/discharge summary of a patient either being offered, accepting or refusing a Quitline referral (e.g. refused Quitline fax referral) (yes, no).
*Acceptance of referral to Quitline.* Acceptance of referral was classified to have occurred if there was any notation in the medical record/discharge summary of a patient accepting a Quitline referral (e.g. Quitline referral faxed) (yes, no).

### Analysis

All analyses were conducted using SAS version 9.4 statistical software [25]. All statistical tests were two tailed with alpha = 0.01.

Descriptive statistics describe the demographic and clinical characteristics of the sample. Chi-square analysis was used to describe such characteristic between the study periods.

Multiple logistic regression models assessed differences in trend between baseline and intervention; intervention and follow-up; and baseline and follow-up periods for each measure of smoking cessation care (seven models in total). Each model included fixed effects for ‘period’ (baseline as referent, intervention and follow-up), time (measured in bi-monthly increments, with six such increments taken during baseline, 12 during intervention and six during follow-up) and the interaction between period and time. The model also included fixed effect for site to account for clustering of observations, as well as the following potential patient-level confounders: patient gender, Aboriginality, age, length of hospital stay, ward class and smoking-related disease. The group-by-time interaction was assessed for significance using the Wald Chi-square test *p* value and was dropped from the model if this did not reach significance. For all models, crude and adjusted odds ratios are presented with 95 % confidence intervals and the adjusted Wald Chi-square test *p* value. Post-hoc comparisons were undertaken to estimate the odds of care between intervention and follow-up. To account for multiple testing, a significance threshold of 0.01 was used to define statistically significant intervention period effects.

#### Inter-rater reliability

Cohen’s kappa was used to assess the inter-rater reliability of the audit of medical records. Strength of agreement was defined as: poor < 0.00, slight 0.00–0.20, fair 0.21–0.40, moderate 0.41–0.60, substantial 0.61–0.80 and almost perfect 0.81–1.00 [30].

### Sample size calculation

It was estimated that approximately 170,000 patients would be discharged by the eight hospitals over the 48-month study period. Twenty percent of discharged patients were estimated to be smokers [18], and 55 % of these were estimated to be nicotine-dependent. Audit of approximately 14 % of all such smokers’ medical records was estimated to provide a total study sample of approximately 2500 nicotine-dependent smokers. Assuming a 50 % prevalence of care at baseline, 80 % power and a 1 % significance level (to account for multiple testing), a difference of 9 % in care was estimated to be detected between baseline and intervention, and between intervention and follow-up periods, and a difference of 11 % in care was estimated to be detected between baseline and follow-up periods.

## Results

### Sample

Over the 4-year study period, 164,252 patients were discharged from the eight selected hospitals. Of these, 17 % (*n* = 6313), 16 % (*n* = 13493) and 16 % (*n* = 6951) were smokers in the baseline, intervention and follow-up periods, respectively. For these smokers, 14.6 % (*n* = 922), 13.6 % (*n* = 1830) and 13.5 % (*n* = 935) of medical records were randomly selected and audited. Slightly more than half (56.2 %; *n* = 2072) of the smokers were nicotine-dependent (*n* = 560 baseline, *n* = 1036 intervention and *n* = 476 follow-up). The sample characteristics are shown in Table 1.

**Table 1:** Sample characteristics

		Period				
Variable		Baseline (*n* = 560)	Intervention (*n* = 1036)	Follow-up (*n* = 476)	*p*	Total (*N* = 2072)^a^
Hospital group	Group A	218 (39 %)	417 (40 %)	203 (43 %)	0.7116	838 (40 %)
	Group B	99 (18 %)	189 (18 %)	87 (18 %)		375 (18 %)
	Group C	243 (43 %)	430 (42 %)	186 (39 %)		859 (41 %)
Gender	Male	318 (57 %)	616 (59 %)	269 (57 %)	0.4328	1203 (58 %)
Aboriginal status	Aboriginal/Torres Strait Islander	58 (10 %)	103 (9.9 %)	48 (10 %)	0.9661	209 (10 %)
Age	18–34	118 (21 %)	189 (18 %)	79 (17 %)	0.1213	386 (19 %)
	35–54	232 (42 %)	505 (49 %)	216 (45 %)		953 (46 %)
	55–74	170 (31 %)	285 (28 %)	152 (32 %)		607 (30 %)
	75+	29 (5.3 %)	52 (5.0 %)	28 (5.9 %)		109 (5.3 %)
Length of stay^a^	4 or less days	363 (66 %)	679 (66 %)	304 (64 %)	0.6190	1346 (65 %)
	5–10 days	122 (22 %)	246 (24 %)	110 (23 %)		478 (23 %)
	11 or more days	64 (12 %)	106 (10 %)	61 (13 %)		231 (11 %)
Ward class^a^	Coronary care	45 (8.2 %)	56 (5.4 %)	16 (3.4 %)	0.0020	117 (5.7 %)
	Medical	156 (28 %)	235 (23 %)	127 (27 %)		518 (25 %)
	Surgical (other than cardiac)	171 (31 %)	337 (33 %)	151 (32 %)		659 (32 %)
	Other	177 (32 %)	403 (39 %)	181 (38 %)		761 (37 %)
Smoking-related disease^a^	Yes	238 (43 %)	382 (37 %)	184 (39 %)	0.0496	804 (39 %)

^a^Data for 17 inpatients missing for length of stay, ward class and smoking-related disease

### Inter-rater reliability

Almost perfect agreement was found for auditing of: offer of inpatient NRT (kappa = 0.94), provision of inpatient NRT (kappa = 0.95) and offer of Quitline referral (kappa = 1.00). Moderate level of agreement was found for: offer of discharge NRT (kappa = 0.513) and provision of discharge NRT (kappa = 0.53) and fair level of agreement for provision of brief advice (kappa = 0.37).

### Provision of smoking cessation care

**Fig. 1:**
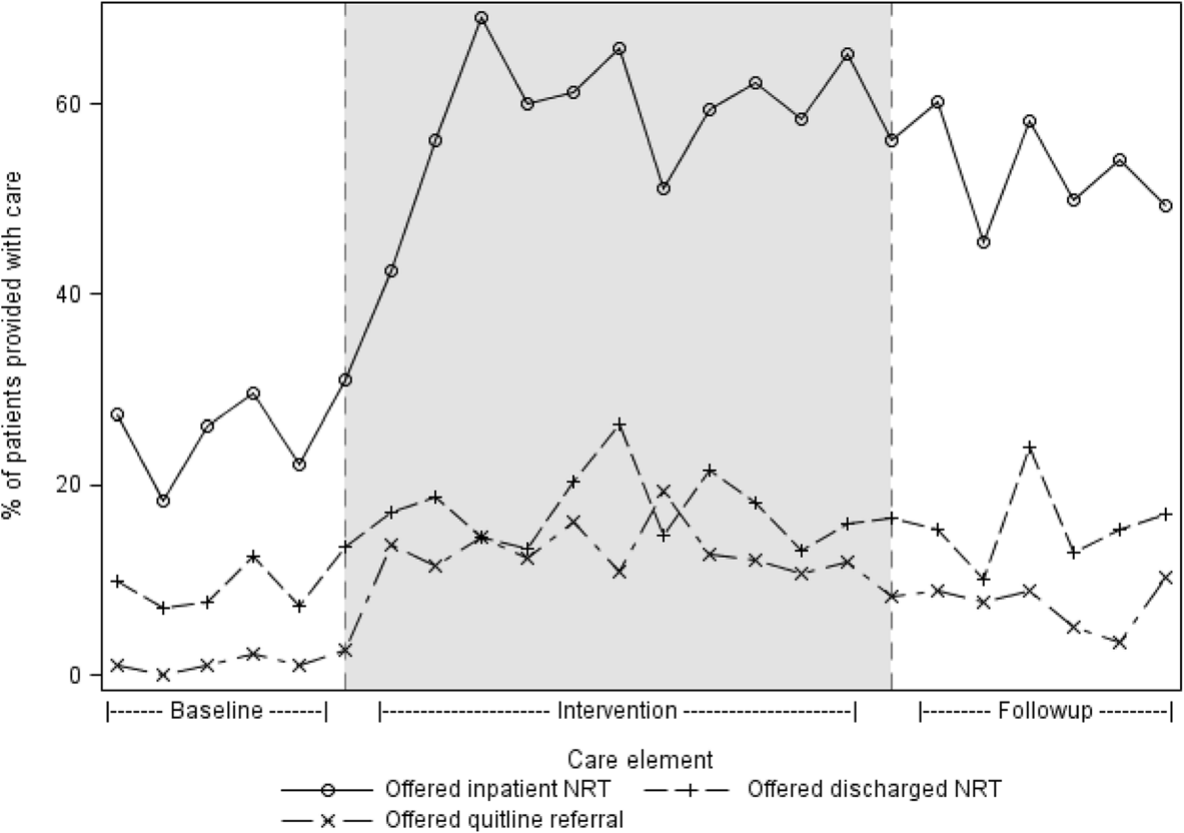
Change in the offer of inpatient and outpatient NRT and Quitline referral

**Fig. 2:**
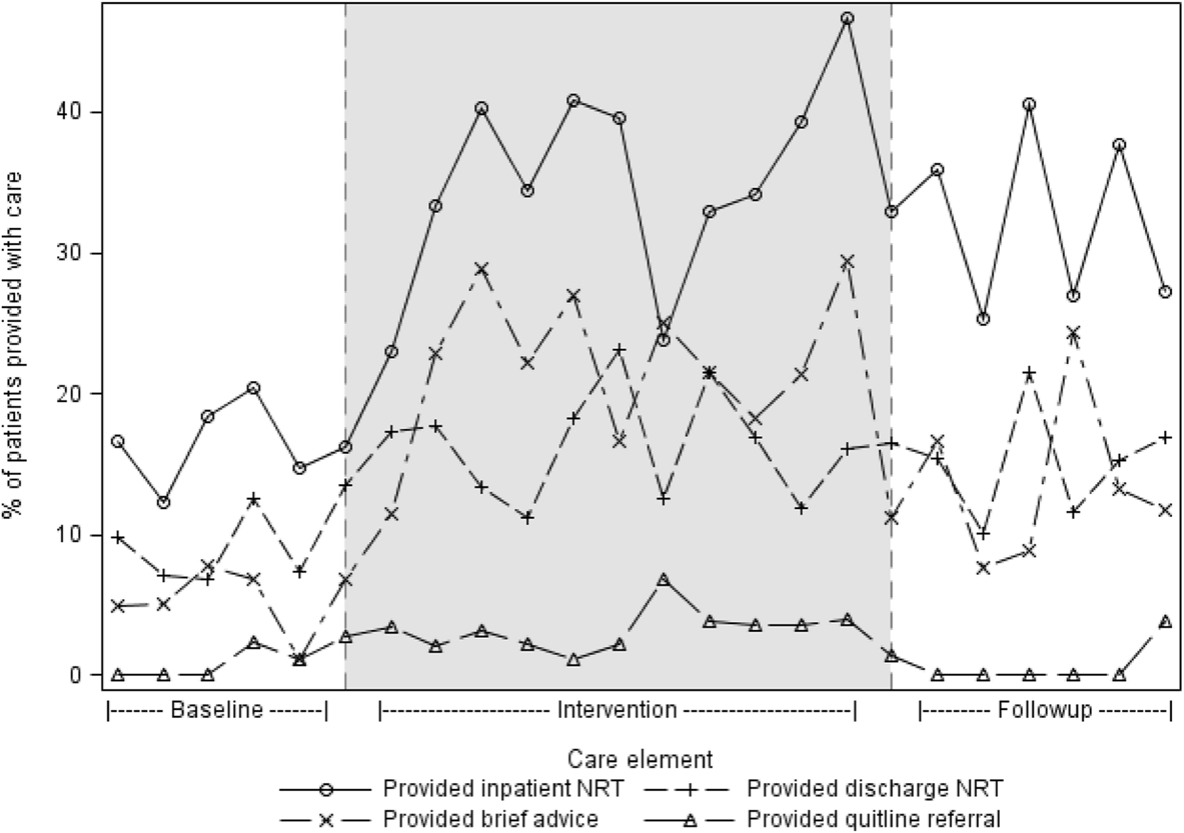
Change in the provision of inpatient NRT, outpatient NRT and brief advice and acceptance of Quitline referral

The proportions of nicotine-dependent patients offered or provided/accepting each care element for each time period are shown in Table 2, Figs. 1 and 2.

**Table 2:** Prevalence of smoking cessation care by period and change in provision

Care element	Time period	*n* (%)	Crude OR (95 % CI)	Adjusted OR (95 % CI)	Adjusted *p* value
Provided smoking care advice	Baseline	30 (5.4 %)	1	1	
	Intervention	222 (21 %)	4.69 (3.16, 6.96)	5.36 (3.51, 8.18)	<0.0001
	Follow-up	65 (14 %)	2.73 (1.74, 4.27)	3.23 (2.03, 5.13)	
Offered inpatient NRT	Baseline	143 (26 %)	1	1	
	Intervention	611 (59 %)	4.21 (3.35, 5.30)	4.33 (3.36, 5.58)	<0.0001
	Follow-up	252 (53 %)	3.27 (2.51, 4.25)	3.50 (2.67, 4.58)	
Provided inpatient NRT	Baseline	92 (16 %)	1	1	
	Intervention	363 (35 %)	2.79 (2.15, 3.62)	2.91 (2.18, 3.89)	<0.0001
	Follow-up	154 (32 %)	2.47 (1.84, 3.33)	2.54 (1.87, 3.45)	
Offered discharge NRT	Baseline	53 (9.5 %)	1	1	
	Intervention	182 (18 %)	2.04 (1.47, 2.82)	2.23 (1.56, 3.20)	<0.0001
	Follow-up	75 (16 %)	1.79 (1.23, 2.60)	1.80 (1.23, 2.64)	
Provided discharge NRT	Baseline	52 (9.3 %)	1	1	
	Intervention	169 (16 %)	1.90 (1.37, 2.65)	2.07 (1.44, 2.99)	0.0004
	Follow-up	72 (15 %)	1.74 (1.19, 2.55)	1.75 (1.18, 2.58)	
Offered Quitline referral	Baseline	7 (1.3 %)	1	1	
	Intervention	134 (13 %)	10.75 (5.11, 22.61)	11.56 (5.47, 24.43)	<0.0001
	Follow-up	35 (7.4 %)	5.65 (2.54, 12.58)	6.27 (2.84, 13.85)	
Accepted Quitline referral	Baseline	5 (0.9 %)	1	1	
	Intervention	32 (3.1 %)	3.22 (1.30, 8.00)	2.58 (1.04, 6.40)	0.0246
	Follow-up	3 (0.6 %)	0.73 (0.19, 2.82)	0.78 (0.22, 2.75)

Between the baseline and intervention periods, there was an increase in all seven measures of smoking cessation care provision of advice to quit smoking (OR 5.36), offer (OR 4.33) and provision of inpatient NRT (OR 2.91), offer (OR 2.23) and provision of NRT on discharge (OR 2.07) and for the offer (OR 11.56) and acceptance of a referral to the Quitline (OR 2.58) (Table 2).

Between the intervention and follow-up periods, there was a slight but statistically significant decrease in three measures of smoking cessation care provision of advice to quit smoking (OR 0.60), offer of a referral to the Quitline (OR 0.54) and acceptance of referral (OR 0.30).

Between the baseline and follow-up periods, there was an increase in six of the seven measures of smoking cessation care advice to quit smoking (OR 3.23), offer (OR 3.50) and provision of inpatient NRT (OR 2.54), the offer (OR 1.80) and provision of NRT on discharge (OR 1.75) and for the offer of a referral to the Quitline (OR 6.27) (Table 2). No change was evident in patient acceptance of referral to the Quitline.

## Discussion

This study suggests that a network-wide practice change intervention increased both the offer and acceptance of multiple forms of smoking cessation care. Notwithstanding the observed increases in care delivery, the most commonly provided form of care (inpatient NRT) was offered to only 53 % of nicotine-dependent patients at follow-up, with the remaining forms of care being offered to no more than 16 %. Even lower proportions of patients were provided or accepted such care.

Despite methodological differences between studies, the magnitude of the observed increases in care delivery are similar, and in some cases greater than those reported in previous research studies (range 6–27 %). A previous meta-analysis reported a pooled risk difference of 16.6 in the delivery of smoking cessation counselling and advice [18]. Similarly, in a controlled study conducted across four hospitals in the same state as the current study, increases in cessation care ranged from 9 to 22 % [17]. Such findings suggest that similar increases in care delivery may be obtained when implementing cessation care across a large number of hospitals as in controlled research studies. In contrast, the observed increases in care delivery are less than the reported increase ranging from 32 to 58 % in the delivery of smoking advice by Joint Commission accredited US hospitals [16]. The extent to which this greater increase is attributable to differences between jurisdictions, in patient inclusion, measurement approach or to intervention approach is unknown.

The finding that the delivery of six of seven elements of care significantly increased compares favourably to increases in a more limited range of smoking cessation care elements in previous studies [17, 18]. Possible explanations for the difference in outcomes between the studies may include a longer intervention length and the inclusion of whole-of- organisation executive leadership in the current study. An ability to increase multiple elements of smoking cessation care is of particular importance as evidence suggests that patient receipt of multiple elements of such care increases the likelihood of stopping smoking [3, 4, 15]. Such findings support the 2012 Joint Commission’s introduction of a broadened core tobacco treatment measurement set. However, the potential for impact of this on the prevalence of patient smoking will be dependent on the number of hospitals that choose to report such data, given the discretionary nature of such reporting [15].

The largest increase in care was found for clinician offer of inpatient NRT (27 %), with variable and much lower increases found for the remaining care elements. Offer of inpatient NRT also showed the largest effect size in the previous study conducted in the same state [17], a finding that suggests that clinicians may be more amenable to medication prescribing than provision of behavioural counselling or provision of follow-up care [31]. Further research is required to identify clinician barriers to the offer of different forms of smoking cessation care.

Fewer patients were provided or accepted each element of care than were offered it. Patient choice to accept offered care is a recognised essential element of quality health care delivery. Nicotine-dependent patients who refused care may have been able to manage their nicotine withdrawal without smoking [12], may have chosen to smoke whilst an inpatient [12], or with regard to discharge NRT or referral to the Quitline, not interested in permanently stopping smoking at that time [10]. The quality of clinician offer of smoking cessation care may also have impacted on patient acceptance of the offer of care. Clinicians express a lack of confidence and skill in the provision of cessation care [14], or a lack of belief that the provision of such care is a part of their role, or is effective [14]. As a large proportion of patients wish to stop smoking [12], and indicate a willingness to accept hospital based cessation care [32], further research is required to identify patient reasons for non-acceptance of offered care, particularly for acceptance of referral to follow-up care (e.g. the Quitline).

Despite the intervention incorporating a wide variety of evidence-based practice-change strategies, a significant proportion of patients were not provided smoking cessation assistance. This suggests that additional strategies may be required to ensure that all nicotine-dependent patients are offered care. Previous reviews and studies have suggested that interventions that directly address hospital systems are more likely to maximise cessation care delivery [17, 19, 33]. For example, the development of computer support systems and the use of smoking cessation care key performance indicators and inclusion of such indicators in hospital accreditation have been suggested to increase the provision of such care [3, 19–21, 34]. Further, such approaches have the potential to address the need for sustainability of improvements in care delivery, a need suggested by the slight decrease in care delivery found in this study for three care elements between the intervention and follow-up periods.

The results of this study should be interpreted in the context of a number of its methodological characteristics. First, the study involved a number of design strengths including: inclusion of a wide spectrum of patient groups, best practice change strategies, addressing multiple elements of smoking cessation care, including measures of both offer and provision of care and independent measurement of care delivery.

Second, a non-controlled study design was selected for pragmatic reasons given the whole-of-system practice change focus of the intervention [35]. As a consequence, the ability to directly attribute the observed increases in care delivery to the intervention is limited. However, the use of a time series design that considered the potential impact of secular trends on the intervention outcome is considered to strengthen the conclusion that it is likely that the intervention had a positive impact on care delivery [36].

Third, not all hospitals that received the intervention were included in the evaluation component of the research due to feasibility. As such, the effectiveness of the intervention within those facilities is unclear. However, the included hospitals were selected to be representative of the hospital profile within the health district. Last, a broad definition for dependence was used to identify nicotine-dependent patients in the medical record, and hence, non-dependent smokers may have been included in the patient sample.

## Conclusions

This study indicated that significant gains can be made in the routine provision of some elements of smoking care in hospitals. However, the findings also suggest that additional care enhancement strategies are required if all nicotine-dependent smokers are to obtain the intended benefits of smoking cessation care guidelines. Further research should investigate the effectiveness of system-based interventions on the provision of evidence-based smoking cessation care.

## Consent

As the study involved a service delivery quality improvement initiative and involved a retrospective audit of de-identified patient medical records, written patient consent was not required by the Hunter New England Human Research Ethics Committee. The audit adhered to the requirements for privacy and confidentiality of patient data and clinical information as required by the New South Wales Information Privacy Act (2002).
